# Hepatic arterial infusion chemotherapy for metastatic colorectal cancer: real-world outcomes of intensification and salvage strategies

**DOI:** 10.1093/oncolo/oyag214

**Published:** 2026-06-04

**Authors:** Annalice Gandini, Federico Lipsich, Jeanne Netter-Coti, Widad Lahlou, Celine Lepere, Xavier Guerra, Olivier Pellerin, Julien Taieb, Tom Boeken, Claire Gallois

**Affiliations:** Institut du Cancer Paris CARPEM, AP-HP Centre, Department of Gastroenterology and Digestive Oncology, Hôpital Européen Georges Pompidou, Paris, 75015, France; SIRIC CARPEM Comprehensive Cancer Center, Paris-Cité University, Paris, 75006, France; Department of Vascular and Oncological Interventional Radiology, Hôpital Européen Georges, Pompidou, Paris, 75015, France; Université Paris Cité, Sorbonne Université, Inserm, Centre de Recherche des Cordeliers, F-75006 Paris, France; Institut du Cancer Paris CARPEM, AP-HP Centre, Department of Gastroenterology and Digestive Oncology, Hôpital Européen Georges Pompidou, Paris, 75015, France; Institut du Cancer Paris CARPEM, AP-HP Centre, Department of Gastroenterology and Digestive Oncology, Hôpital Européen Georges Pompidou, Paris, 75015, France; Institut du Cancer Paris CARPEM, AP-HP Centre, Department of Gastroenterology and Digestive Oncology, Hôpital Européen Georges Pompidou, Paris, 75015, France; SIRIC CARPEM Comprehensive Cancer Center, Paris-Cité University, Paris, 75006, France; Department of Vascular and Oncological Interventional Radiology, Hôpital Européen Georges, Pompidou, Paris, 75015, France; SIRIC CARPEM Comprehensive Cancer Center, Paris-Cité University, Paris, 75006, France; Department of Vascular and Oncological Interventional Radiology, Hôpital Européen Georges, Pompidou, Paris, 75015, France; Institut du Cancer Paris CARPEM, AP-HP Centre, Department of Gastroenterology and Digestive Oncology, Hôpital Européen Georges Pompidou, Paris, 75015, France; Université Paris Cité, Sorbonne Université, Inserm, Centre de Recherche des Cordeliers, F-75006 Paris, France; SIRIC CARPEM Comprehensive Cancer Center, Paris-Cité University, Paris, 75006, France; Department of Vascular and Oncological Interventional Radiology, Hôpital Européen Georges, Pompidou, Paris, 75015, France; Institut du Cancer Paris CARPEM, AP-HP Centre, Department of Gastroenterology and Digestive Oncology, Hôpital Européen Georges Pompidou, Paris, 75015, France; Université Paris Cité, Sorbonne Université, Inserm, Centre de Recherche des Cordeliers, F-75006 Paris, France

**Keywords:** metastatic colorectal cancer, hepatic arterial infusion chemotherapy, liver metastases, intensification treatment, salvage therapy

## Abstract

**Background:**

Hepatic arterial infusion chemotherapy (HAIC) is a valuable option in patients with liver-dominant metastatic colorectal cancer (mCRC) but remains underutilized due to limited data.

**Methods:**

We conducted a retrospective study of mCRC patients treated with HAIC in an expert center between 2010 and 2024. Patients were grouped according to treatment setting: intensification (INT; 1st/2nd line) and salvage (SALV; ≥3rd line).

**Results:**

Among 213 patients, 99 received INT-HAIC and 114 SALV-HAIC. SALV patients had worse baseline features, including more Eastern Cooperative Oncology Group performance status (ECOG PS ≥ 2; 16% vs 4%), *RAS* mutation (55% vs 45%), extra-hepatic disease (45% vs 23%), liver burden >50% (65% vs 40%), and prior intravenous (IV) oxaliplatin progression (41% vs 16%). Oxaliplatin was the main agent used (81% INT, 78% SALV). Objective response and disease control rate were 51%/77% (INT) and 32%/60% (SALV). Median progression-free survival (PFS), hepatic PFS, and overall survival were 7.6, 9, and 23 months (INT) and 3.7, 5.7, and 12 months (SALV). Prior IV oxaliplatin progression was associated with poorer outcomes of oxaliplatin-HAIC in the INT setting. Radical liver treatment followed HAIC in 28% (INT) and 7% (SALV). Grade 3–4 adverse events occurred in 42.7% and catheter complications in 26.7%. Concomitant antiangiogenic therapy was associated with a higher rate of catheter-related complications (44% vs 22%) and remained associated after adjustment for clinical covariates.

**Conclusions:**

HAIC demonstrates promising efficacy and manageable toxicity in liver-dominant mCRC when delivered in expert multidisciplinary centers, although careful patient selection and caution with concomitant antiangiogenic therapy are warranted.

Implications for Practice Hepatic arterial infusion chemotherapy (HAIC) represents an effective therapeutic option for patients with liver-dominant metastatic colorectal cancer. This real-world analysis supports its use both as treatment intensification and as salvage therapy, with meaningful response rates and the potential to enable secondary radical liver treatments, particularly in earlier lines. Careful patient selection is essential, as prior progression on intravenous oxaliplatin predicts limited benefit from oxaliplatin-based HAIC in the intensification setting. Toxicity and catheter-related complications are manageable but require dedicated expertise, underscoring the importance of referral to specialized centers, and caution is warranted when combining HAIC with concomitant antiangiogenic therapy due to an increased risk of catheter-related complications.

## Introduction

Colorectal cancer (CRC) is the third most common cancer worldwide^1^ and approximately 20% of cases are diagnosed at an advanced stage.^2^ The liver is the most frequent metastatic site and is associated with poorer survival outcomes.^3^ In patients with unresectable liver metastases, the standard of care is cytotoxic chemotherapy combined with targeted agents, with disease control as the primary goal and downstaging to enable potentially curative resection as a key objective.^4^ In patients with liver-only or “liver-dominant” metastatic CRC (mCRC), liver-directed intra-arterial therapies, such as hepatic arterial infusion chemotherapy (HAIC), represent an alternative treatment strategy.^4^ This approach is supported by a strong anatomical rationale, as liver metastases are predominantly vascularized by the hepatic artery, while liver parenchyma by the portal vein,^5^ allowing higher intra-tumoral drug concentrations with potentially reduced systemic toxicity.^6^

Although HAIC has shown promising results, evidence supporting its use in unresectable mCRC remains limited and is largely derived from small phase II studies conducted in selected populations. The OPTILIV single-arm trial demonstrated the feasibility of combining HAIC with systemic therapy in pretreated patients, with encouraging conversion-to-resection rates.^7^ The first randomized evidence came from the phase II SULTAN trial, which planned to randomize 140 patients with liver-only unresectable mCRC after induction chemotherapy to receive oxaliplatin HAIC plus systemic treatment or standard of care. The study closed early due to slow accrual, enrolling only 26 patients. Despite the extremely limited sample size, the complete resection rate was 64% (7/11) in HAIC arm vs 22% (2/9) in the standard arm (*P* = .09), suggesting potential efficacy.^8^^,^^9^ Similarly, in the first-line setting, the phase II CHOICE trial reported a median progression-free survival (mPFS) of 17.9 months and median overall survival (mOS) of 46.3 months in 35 patients treated with oxaliplatin-HAIC associated with intravenous (IV) 5FU plus cetuximab.^10^

In contrast, the PACHA-01 randomized phase II trial evaluated HAIC in the adjuvant setting following liver metastasis resection and demonstrated an improvement in hepatic relapse-free survival.^11^ While representing the highest level of randomized evidence to date, this study addresses a distinct clinical scenario and does not inform the role of HAIC in unresectable metastatic disease.

Given these limitations and the absence of phase III comparative data, the role of HAIC in unresectable colorectal liver metastases remains to be better defined. We have, therefore, conducted a large retrospective study aiming to assess the efficacy and safety of HAIC in patients with liver-dominant mCRC treated in a high-volume expert center and to identify clinical features associated with improved outcomes.

## Methods

### Patients

All consecutive patients with histologically confirmed mCRC who received at least one cycle of HAIC at the Hôpital Européen Georges Pompidou between April 2010 and November 2024 were retrospectively included.

### Data collection

Data on tumor, prior treatments, HAIC protocols, toxicity, treatment response (as per standard clinical assessments), and survival were collected in a pseudonymized database.

Baseline CT scans prior to HAIC were re-reviewed by a board-certified radiologist to assess liver tumor burden. If multiple HAIC treatment lines were administered, only the first was included in the analysis. In patients who switched HAIC regimen due to toxicity without evidence of progressive disease (PD), PFS and hepatic PFS (HPFS) were calculated from HAIC initiation to PD under the subsequent HAIC regimen. For patients who switched IV systemic regimens due to extra-hepatic progression, while continuing the same HAIC protocol, PFS events were defined as the first occurrence of extra-hepatic progression, whereas HPFS events were defined as the hepatic progression. Adverse events (AEs) were categorized as catheter-related complications or chemotherapy-related toxicities. Each AE was graded according to the Common Terminology Criteria for Adverse Events (CTCAE) version 5.0.

Data were collected from electronic medical records after approval by the institutional review board (CERAPHP Center, IRB No: IORG0010044). The study was conducted in accordance with the Declaration of Helsinki.

### Statistical analysis

Median (interquartile range) values and proportions (percentage) were used for continuous and categorical variables, respectively. Median and proportions were compared using the Wilcoxon-Mann-Whitney test and the chi^2^-test (or Fisher’s exact test, if appropriate), respectively. A Cochran–Armitage trend test was used to assess linear trends across ordered categories.

Objective response rate (ORR) was defined as the proportion of patients achieving a complete response (CR) or partial response (PR). Disease control rate (DCR) included CR, PR, or stable disease (SD).

PFS was defined as the time between the start of HAIC and tumor progression at any site or death, whichever occurred first. HPFS was defined as the time between the start of HAIC and the first documented progression in the liver. OS was defined as the time between HAIC start and death from any cause. Patients known to be alive were censored at the date of their last follow-up. PFS, HPFS, and OS were estimated using the Kaplan–Meier method. Follow-up was calculated using the reverse Kaplan–Meier method.

Efficacy analyses were performed separately for two subgroups: the “HAIC as intensification treatment” group, defined by HAIC administration in the first or second line, and the “HAIC as salvage treatment” group, defined by HAIC administration in the third line or beyond.

Factors associated with PFS and OS were investigated using univariate and multivariable Cox proportional hazards models. Variables with a *P* value <0.1 in univariate analysis, or deemed clinically relevant, were included in the multivariable models.

For safety analyses, if a patient switched HAIC regimen due to toxicity, only the catheter-related complications from the second regimen were added to those from the first.

All statistical analyses were conducted using R software version 3.2 (R Development Core Team, Vienna, Austria; http://www.r-project.org). Two-sided *P* values <.05 were considered statistically significant.

## Results

### Patient characteristics

**Figure 1.**
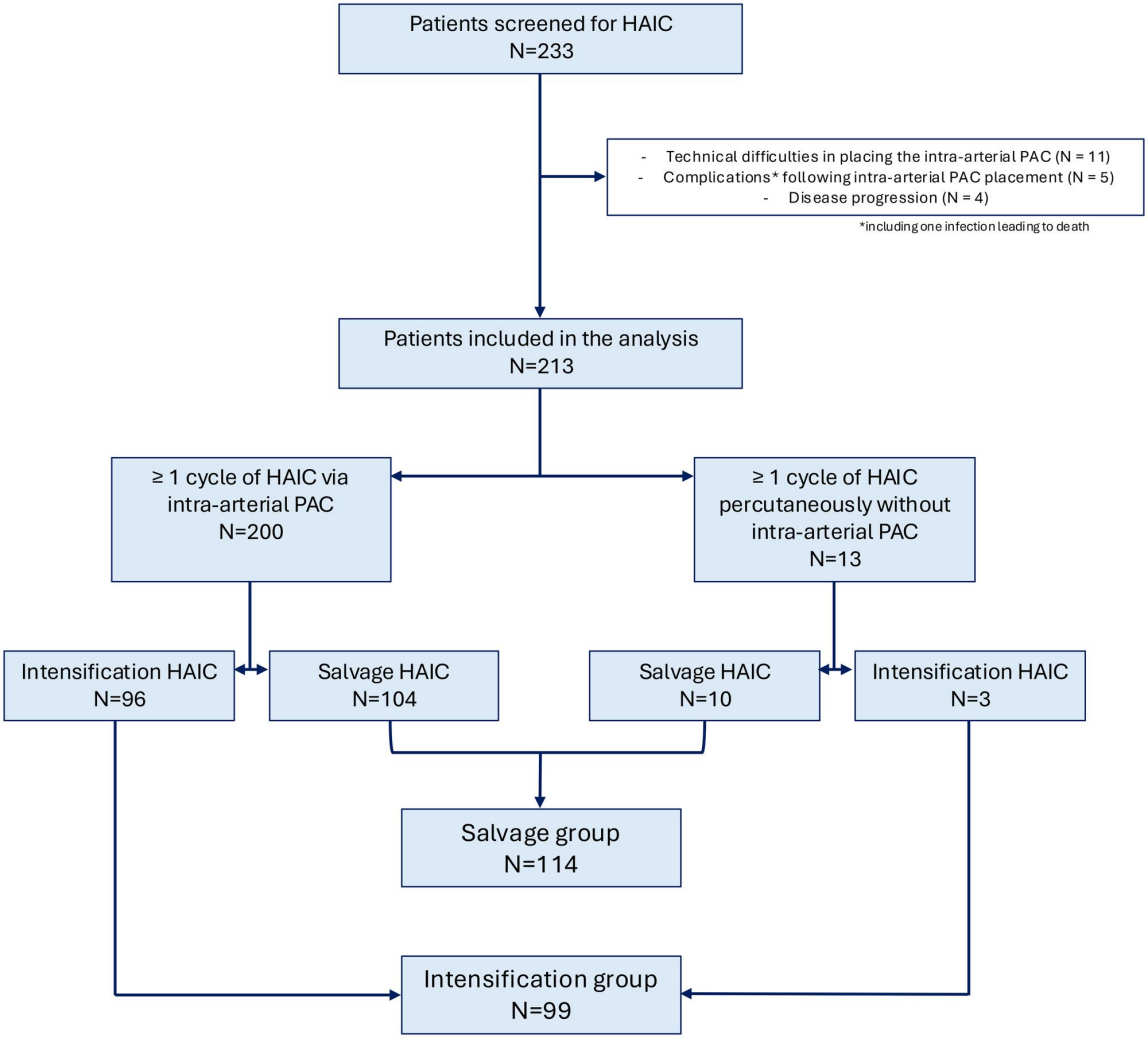
Study flowchart. Abbreviations: HAIC, hepatic arterial infusion chemotherapy; PAC, Port-a-Cath. ^a^Including one infection leading to death.

HAIC was recommended for 233 patients after evaluation by a multidisciplinary tumor board. Among them, 20 patients were excluded from the analysis because they ultimately did not receive HAIC, due to technical difficulties (*N* = 11), complications following intra-arterial Port-a-Cath (PAC) placement (*N* = 5) or disease progression (*N* = 4). For anatomical or logistic reasons, 13 patients received percutaneous hepatic arterial chemotherapy without intra-arterial PAC placement; instead, treatment was administered directly via an infusion pump during hospitalization (Figure 1).

The final cohort, consisting of patients who received at least one cycle of HAIC, included 213 patients: 99 patients in the intensification group and 114 in the salvage group.

There were no significant differences between the intensification and salvage groups regarding age (median age: 59 vs 61 years), sex (female: 38% vs 39%), timing of metastatic disease (synchronous in 86% vs 83%), and *RAS* mutation status (45% vs 55%). Compared with the intensification group, patients in the salvage group more frequently had an ECOG performance status (ECOG PS) of 2-3 (16% vs 4.2%, *P* = .003), extra-hepatic disease (45% vs 23%, *P* = .001), ≥2 extra-hepatic sites (22% vs 9%, *P* = .004), liver burden >50% (65% vs 40%), and prior progression on oxaliplatin IV (41% vs 16%, *P* < .001). Most patients in the intensification group received HAIC as second-line therapy (83% of cases), whereas those in the salvage group predominantly received HAIC as third (64%) and fourth line (22%) (Table 1). Thirteen patients in the intensification group (13%) were treated in the first line within the OSCAR trial^12^ during the study period.

**Table 1:** Patients’ characteristics in the intensification and salvage settings.

	Intensification *N* = 99^a^	Salvage *N* = 114^a^	*p* value^b^
**Median age**	59 (51, 68)	61 (52, 70)	.084
**Age > 65 years**	32 (32%)	41 (36%)	.7
**Sex**			>.9
** Female**	38 (38%)	44 (39%)	
** Male**	61 (62%)	70 (61%)	
**ECOG-PS**			**.003**
** 0**	36 (38%)	21 (19%)	
** 1**	56 (58%)	71 (65%)	
** 2**	4 (4.2%)	15 (14%)	
** 3**	0 (0%)	2 (1.8%)	
** Unknown**	3	5	
**Primary tumor location**			.2
** Distal colon**	80 (82%)	80 (73%)	
** Proximal colon**	18 (18%)	29 (27%)	
** Unknown**	1	5	
**Metastatic disease onset**			.4
** Metachronous**	14 (14%)	19 (17%)	
** Synchronous**	85 (86%)	94 (83%)	
** Unknown**	0	1	
**Primary tumor surgery**	71 (72%)	85 (75%)	.6
**dMMR**	1 (1.2%)	1 (1.1%)	>.9
** Unknown**	16	22	
***RAS* mutated**	40 (45%)	58 (55%)	.2
** Unknown**	10	9	
***BRAF* mutated**	8 (9.9%)	2 (2.6%)	.1
** Unknown**	18	37	
**Extrahepatic disease**	23 (23%)	51 (45%)	**.001**
**Number of extrahepatic metastatic sites**			**.004**
** 1**	21 (91%)	40 (78%)	
** 2**	2 (9%)	9 (18%)	
** 3**	0 (0%)	2 (4%)	
**Liver burden**			**.01**
** 0%-25%**	26 (31%)	17 (17%)	
** 26%-50%**	24 (29%)	19 (18%)	
** 51%-75%**	16 (19%)	31 (30%)	
** 76%-100%**	18 (21%)	36 (35%)	
** Unknown**	15	11	
**No. of prior treatments**			**<.001**
** 0**	17 (17%)	0 (0%)	
** 1**	82 (83%)	0 (0%)	
** 2**	0 (0%)	73 (64%)	
** 3**	0 (0%)	25 (22%)	
** 4**	0 (0%)	9 (7.9%)	
** 5**	0 (0%)	5 (4.4%)	
** 7**	0 (0%)	2 (1.8%)	
**Prior adjuvant HAIC**	5 (5.1%)	1 (0.9%)	.1
**Prior progression on oxaliplatin IV**	16 (16%)	47 (41%)	**<.001**

Significant *P* values are in bold.

Abbreviations: dMMR, deficient mismatch repair; ECOG PS, Eastern Cooperative Oncology Group Performance Status.

a Median (Q1, Q3); *n* (%).

b Wilcoxon rank sum test for continuous variables; Fisher’s exact test for categorical variables.

Regarding subsequent lines of HAIC, 46 patients (22%) received a second line of HAIC.

### Treatment regimens

HAIC regimens included oxaliplatin-HAIC (*N* = 169, 79%), 5FU-HAIC (*N* = 37, 17%), mitomycin-HAIC (*N* = 4, 1.9%), or other (*N* = 3, 1.4%).

IV chemotherapy was associated concomitantly in 99% of cases (*N* = 211), most commonly 5FU IV or FOLFIRI IV. 74 patients (35%) received a concomitant IV monoclonal antibody (MoAb) (Table S1). The median number of HAIC cycles was 6 (range 1-47). A total of 29 patients (14 and 15 in the intensification and salvage groups, respectively) switched HAIC regimen due to toxicity, without evidence of PD. Most patients switched from oxaliplatin-based HAIC to 5FU-based HAIC (*n* = 27), while two patients switched to mitomycin-based HAIC. A detailed patient-level description of toxicities leading to treatment switch is provided in Table S2.

### Outcomes

#### Best response rate

The DCR was 67.6% in the overall population: 77% (95% CI, 74.3-90.5) in the intensification group and 67% (95% CI: 57.3-76.3) in the salvage group. The ORR was 40.6% in the overall population, 51% (95% CI: 39.9-61.2) in the intensification group and 32% (95% CI: 22.8-41.7%) in the salvage group. Oxaliplatin-HAIC was associated with higher ORR across all subgroups (Table 2).

**Table 2:** Best response rates in the overall population and according to HAIC setting (intensification vs salvage) and HAIC regimen.

	Overall population (%)	Intensification (%)	Salvage (%)
**ORR**	41	51	32
** 5FU-HAIC (*N* = 37)**	27	37	19
** Oxaliplatin-HAIC (*N* = 148)**	45	54	35
**DCR**	68	77	60
** 5FU-HAIC (*N* = 37)**	57	62	52
** Oxaliplatin-HAIC (*N* = 148)**	70	90	71

The number of patients (*N*) for each regimen refers to patients with available radiological assessment; best response data were missing for 21 patients treated with oxaliplatin-HAIC. Significant *P* values are in bold.

Abbreviations: DCR, disease control rate; HAIC, hepatic arterial infusion chemotherapy; ORR, objective response rate.

A curative-intent liver treatment (surgery and/or locoregional therapy) was performed in 28% of patients in the intensification group and 7% in the salvage group.

#### Survival outcomes

**Figure 2.**
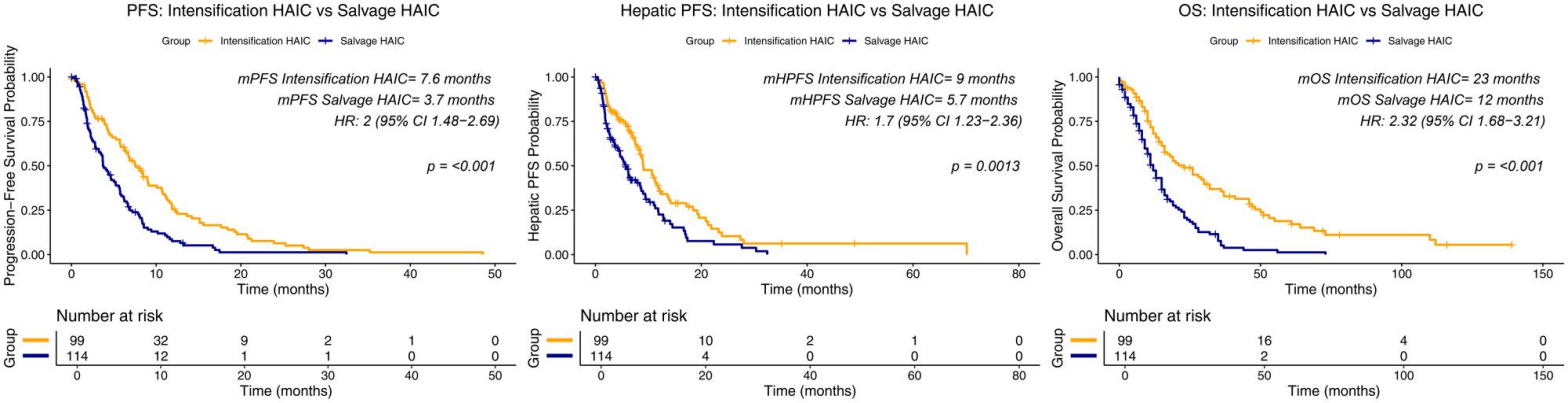
Kaplan-Meier curves for PFS, hepatic PFS, and OS in patients treated with HAIC in the intensification group and in the salvage group. Abbreviations: PFS, progression-free survival; OS, overall survival; HAIC, hepatic arterial infusion chemotherapy; HR, hazard ratio.

After a median follow-up of 72 months, patients who received HAIC as intensification therapy in the first or second line had a mPFS of 7.6 months (95% CI: 6-10), a mHPFS of 9 months (95% CI: 8-12), and a mOS of 23 months (95% CI: 16-32). Patients treated with HAIC as salvage therapy had a mPFS of 3.7 months (95% CI: 3-5), a mHPFS of 5.7 months (95% CI: 4-8.6), and a mOS of 12 months (95% CI: 10-15) (Figure 2).

As salvage treatment, in the subgroup of patients treated in the third line (*N* = 73), the ORR was 36.5%, with mPFS of 3.7 months, mHPFS of 7.9 months, and mOS of 13 months. In patients treated beyond the third line (*N* = 41), the ORR was 23.7%, mPFS was 1.8 months, mHPFS was 3.4 months, and was mOS 10 months.

Tables S3 and S4 show the factors associated with PFS and OS in univariate analyses among patients treated in the intensification or salvage setting.

In the intensification group, multivariable analysis showed that *BRAF* mutation remained significantly associated with shorter PFS (adjusted hazard ratio [adjHR], 8.8; 95% CI, 1.09-71.2; *P* = .04), whereas the use of oxaliplatin-based HAIC was protective (adjHR, 0.23; 95% CI, 0.08-0.69; *P* = .008), and the only factor independently associated with shorter OS was prior progression on IV oxaliplatin (adjHR, 4.9; 95% CI, 1.6-14.67; *P* = .004), regardless of other prognostic factors, including liver tumor burden (Table S3).

In the salvage group, multivariable analysis identified ECOG-PS >1 (adjHR, 2.5; 95% CI, 1.05-5.96; *P* = .04) and CEA level >158 ng/mL (adjHR, 2.28; 95% CI, 1.14-5.57; *P* = .02) as factors independently associated with shorter PFS. Factors independently associated with shorter OS were liver tumor burden >75% (adjHR, 2.6; 95% CI, 1.1-6.05; *P* = .03) and elevated CA19.9 levels (adjHR, 2.5; 95% CI, 1.37-4.6; *P* = .003) (Table S4).

#### Efficacy outcomes based on prior progression on oxaliplatin IV in patients receiving oxaliplatin-HAIC

Among the 80 patients in the intensification group treated with oxaliplatin-HAIC, 10 (12.5%) had previously progressed on oxaliplatin IV, with a median oxaliplatin-free interval of 1 month [IQR 0-2.5]. Among the 89 patients in the salvage group treated with oxaliplatin-HAIC, 38 (42.7%) had previously progressed on oxaliplatin IV, with a median oxaliplatin-free interval of 7 months [IQR 4-12.8].

**Figure 3.**
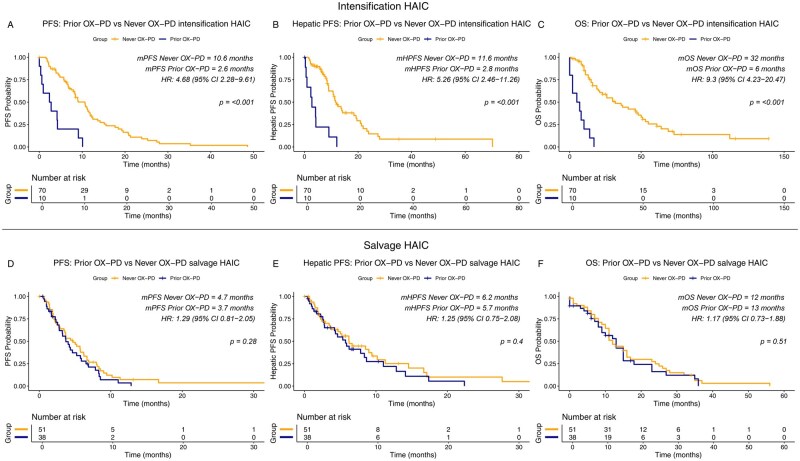
Kaplan-Meier curves for PFS, hepatic PFS, and OS according to prior progression on oxaliplatin IV in patients treated with oxaliplatin-HAIC. Panels A-B-C show outcomes in the intensification HAIC group (A: PFS; B: HPFS; C: OS), while panels D-E-F show outcomes in the salvage HAIC group (D: PFS; E: HPFS; F: OS). Abbreviations: HAIC, hepatic arterial infusion chemotherapy; PFS, progression-free survival; OS, overall survival; OX-PD, oxaliplatin progression; HR, hazard ratio.

In the intensification group, prior progression on oxaliplatin IV was associated with significantly shorter survival (*P* < .001): mPFS 2.6 vs 10.6 months, mHPFS 2.8 vs 11.6 months, and mOS 6 vs 32 months. By contrast, prior progression on oxaliplatin IV was not associated with decreased PFS, HPFS, or OS in the salvage group (Figure 3).

### Safety

**Figure 4.**
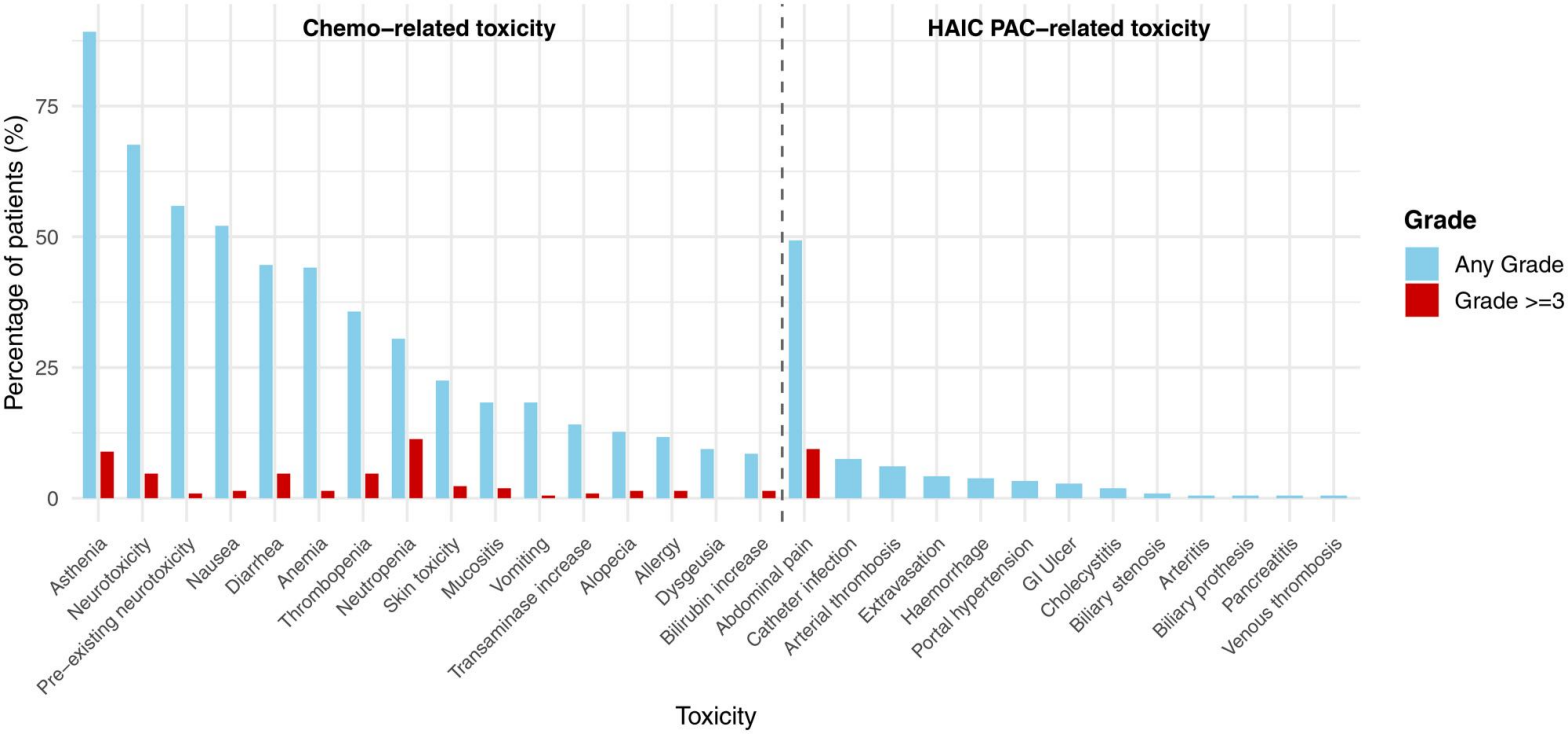
Rates of chemotherapy-related adverse events and PAC-related complications in the overall population. Abbreviations: HAIC, hepatic arterial infusion chemotherapy; PAC, Port-a-Cath.

The most common adverse events (AEs) of any grade related to chemotherapy administration were asthenia (94%), neurotoxicity (71%), nausea (55%), and abdominal pain (52%), most of which were grade 1 or 2. The overall rate of grade 3-4 AEs was 42.7%, with the most frequent being neutropenia (12%), abdominal pain (10%), and asthenia (9%) (Figure 4).

The rate of catheter-related complications, excluding abdominal pain, was 26.7% (*N* = 57), leading to permanent discontinuation of HAIC in 54% of these cases (*N* = 31). These complications included PAC infection (7.5% of all patients), arterial thrombosis (6.1%), extravasation from the arterial PAC (4.2%), hemorrhage (3.7%), portal hypertension (3%), gastrointestinal ulcers (3%), cholecystitis (2%), biliary stenosis (1%), venous thrombosis (0.5%), arteritis (0.5%), and pancreatitis (0.5%) (Figure 4). No treatment-related deaths occurred. The rate of catheter-related complications was higher among patients receiving concomitant antiangiogenic therapy (21/48, 44% vs 36/165, 22%; *P* = .005), particularly vascular complications (thrombosis, arteritis, and hemorrhage) (10/48 = 21% vs 11/165 = 7%; *P* = .01). In multivariable analysis adjusting for prior antiangiogenic exposure, performance status, and HAIC treatment setting, concomitant antiangiogenic therapy remained independently associated with an increased risk of catheter-related complications (adjusted OR 2.50, 95% CI 1.24-5.05; *P* = .01) (Table S5).

Although abdominal pain was related to HAIC, it was not considered a catheter-related complication. It occurred more frequently in patients who received concomitant MoAb (34/48, 71%) than in those who did not (71/165, 43%; *P* = .0009). Among patients treated with oxaliplatin-HAIC, 53.1% experienced abdominal pain, compared with 43.2% of those receiving 5FU-HAIC (*P* = .37). The incidence of abdominal pain did not increase with higher liver tumor burden (Cochran–Armitage trend test, *P* = .9).

## Discussion

In this retrospective observational study, we analyzed data from 213 patients who received HAIC for liver-dominant mCRC across different lines of treatment and compared outcomes according to treatment setting: intensification vs salvage. Patients were well distributed across liver tumor burden categories (0%-25%, 25%-50%, 50%-75%, and >75%), ensuring adequate representation of subgroups.

HAIC in the intensification group was associated with a high ORR of 51% and prolonged survival (mPFS 7.6 months, mHPFS 9 months, mOS 23 months). This high response rate translated into 28% of patients undergoing secondary liver surgery or locoregional treatment with curative intent, a key objective in mCRC management. However, this relatively high rate of locoregional intervention should be interpreted with caution. Data on treatment intent and baseline resectability status at HAIC initiation were not systematically collected, limiting our ability to distinguish between true conversion strategies and purely palliative approaches. After adjustment for classical prognostic factors, *BRAF* mutation, prior progression on IV oxaliplatin, and the use of a non-oxaliplatin HAIC regimen were independently associated with poorer outcomes.

These results appear consistent with previously reported outcomes of HAIC-based strategies in the literature, including retrospective and prospective studies in similar clinical settings. For example, Boileve et al. reported in 89 patients treated with oxaliplatin-HAIC combined with systemic chemotherapy and targeted therapy—mostly in second or third line—an ORR of 42%, a resection rate of 27%, a median PFS of 9 months, and a median OS of 20 months.^13^ Similarly, the CHOICE phase II study and the phase I study by Kemeny et al. have reported high response rates and substantial conversion-to-resection rates with HAIC-based approaches in selected patients.^10^^,^^14^

In the salvage setting, survival outcomes observed in our cohort are in the range of those reported in contemporary third-line treatment studies in mCRC, including trifluridine–tipiracil plus bevacizumab, as evaluated in the SUNLIGHT trial.^15^ Our population, however, differs notably from clinical trial cohorts, as it reflects a real-world setting enriched in patients with liver-only or liver-dominant disease, a subgroup generally associated with poorer prognosis. In this setting, HAIC was associated with an ORR of 36.5% in third-line patients and 23.7% in patients treated beyond the third line, reflecting antitumor activity in a heavily pretreated population. The observed activity of HAIC in this setting further supports its feasibility in heavily pretreated patients, although these results should be interpreted as descriptive and hypothesis generating.

These high response rates across treatment lines support a potential role for HAIC for patients with liver-dominant disease and high hepatic tumor burden, where prognosis is primarily determined by liver involvement.

Interestingly, after adjustment for prognostic factors, high liver tumor burden was not associated with shorter PFS or HPFS, particularly in patients with >50% or >75% hepatic involvement. Moreover, these patients with extensive liver involvement did not experience increased HAIC-related toxicity.

Despite some imbalance in subgroup sizes, oxaliplatin-based HAIC appeared more effective than 5FU-based HAIC in terms of ORR and it was an independent favorable prognostic factor for PFS in the intensification setting. It should therefore be preferred when feasible. However, our findings suggest that oxaliplatin-based HAIC in the intensification setting is unlikely to confer benefit when administered immediately after progression on IV oxaliplatin, and its use in this setting should be discouraged. These findings are likely explained by differences in the oxaliplatin-free interval between treatment settings. In our cohort, patients in the intensification group were re-exposed to oxaliplatin shortly after prior progression, with a median interval of only 1 month, whereas patients in the salvage group had a substantially longer interval (median 7 months). This temporal difference likely reflects distinct biological contexts, with early re-exposure favoring cross-resistance, while a longer drug-free interval may allow partial re-sensitization. In this regard, a re-challenge strategy with oxaliplatin-HAIC may be reasonable only in carefully selected patients with a sufficiently long oxaliplatin-free interval. The small sample size of this subgroup prevents definitive conclusions, and further studies are warranted to assess whether 5FU-based HAIC may be more effective after oxaliplatin progression.

Regarding concomitant IV MoAb, its use was not identified as a favorable prognostic factor in multivariable analysis in either setting, likely owing to the limited sample size (*n* = 74 in both groups) and treatment heterogeneity. In line with our findings, Cercek et al. reported that concomitant bevacizumab with FUDR-HAIC did not improve survival or response rate.^16^ The use of concomitant anti-EGFR therapy (cetuximab) was evaluated in the phase II single-arm CHOICE and OPTILIV trials, with encouraging efficacy and no increase in anti-EGFR-related toxicities.^7^^,^^10^

In terms of safety, HAIC appeared feasible in this real-world cohort, although not without risk. HAIC-specific toxicities were observed in a substantial proportion of patients, including abdominal pain in approximately half of cases, as well as catheter-related complications that could impact overall management, prognosis, and quality of life. These findings underscore the importance of conducting HAIC in specialized centers with dedicated interventional radiology and multidisciplinary expertise, as well as the need for careful patient selection and close monitoring. Despite PAC-related biliary toxicity being lower than those reported with FUDR-HAIC regimen,^14^^,^^17–19^ we demonstrated for the first time that concomitant antiangiogenic therapy was associated with significantly higher incidence of all PAC-related AEs (44% vs 22%, *P* = .005). This association remained significant after adjustment for key clinical variables, including prior antiangiogenic exposure, performance status, and HAIC treatment setting, supporting a potential independent effect of antiangiogenic agents on PAC-related AEs and highlighting the need for caution. Similar findings have been reported with bevacizumab and FUDR-HAIC, where increased biliary toxicity was observed.^16^

The main strengths of our study include its large real-world cohort of 213 mCRC patients, its evaluation on HAIC across different treatment settings, and the centralized review of all baseline CT scans to quantify liver tumor burden. This parameter was integrated into predictive survival models and analyzed for its association with toxicity—an approach not previously explored. Nonetheless, several limitations must be acknowledged, including the retrospective and monocentric design, and the relatively small size of certain subgroups, which limit statistical power and preclude detailed subgroup analyses. It should also be noted that a small subset of patients was enrolled in a prospective first-line clinical trial during the study period, representing 6% of the overall cohort and 13% of the intensification group. Given these limited proportions, their inclusion is unlikely to have materially affected the overall results. Finally, in the absence of prospective comparative data with standard IV chemotherapy, these findings do not support the routine use of HAIC and support its evaluation within prospective clinical trials, which remain essential to define its role in clinical practice.

In conclusion, HAIC represents a feasible therapeutic option, although associated with procedure-related risks, and may offer clinical benefit for patients with unresectable liver-only or liver-dominant disease when performed in specialized centers with multidisciplinary expertise. In the intensification setting, high response and resection rates support further investigation of this strategy in dedicated randomized clinical trials (eg, the ongoing OSCAR trial^12^), while prior progression on oxaliplatin was associated with poorer outcomes, underscoring the importance of patient selection based on the oxaliplatin-free interval. In the salvage setting, HAIC appears particularly promising for achieving disease control, and prior oxaliplatin progression did not adversely impact outcomes. High liver tumor burden was not associated with poorer outcomes or increased toxicity, while concomitant antiangiogenic therapy was associated with an increased risk of catheter-related complications, supporting caution when combining these strategies.

## Supplementary Material

oyag214_Supplementary_Data

## Data Availability

The data supporting the findings of this retrospective single-center study are available from the corresponding author upon reasonable request. The data are not publicly available due to patient confidentiality and ethical restrictions.
