# Supplementary material for: Hepatic arterial infusion chemotherapy for metastatic colorectal cancer: real-world outcomes of intensification and salvage strategies
Source: Oncologist. 2026 Jun 4;31(8):oyag214. doi: 10.1093/oncolo/oyag214 (PMC13375640; doi:10.1093/oncolo/oyag214)
Supplement: oyag214_Supplementary_Data [file oyag214_supplementary_data.docx]

**Supplementary Table 1. Treatment regimens in the intensification and in the salvage setting*.*** Significant p-values are highlighted in bold. Acronyms: IV=intravenous; CT=chemotherapy; MoAb=monoclonal antibody; 5FU=5 fluorouracil; FOLFOX= 5FU+oxaliplatin; FOLFIRI=5FU+irinotecan; FOLFIRINOX=5FU+irinotecan+oxaliplatin.

|  | **Intensification**  N = 99^1^ | | **Salvage**  N = 114^1^ | | **p-value**^2^ | |  |  |
| --- | --- | --- | --- | --- | --- | --- | --- | --- |
| **IV-CT** | | | 99 (100%) | | 112 (98%) | | 0.5 | |
| **IV-CT regimen** |  | |  | | 0.2 | |  |  |
| 5FU | 51 (52%) | | 68 (61%) | |  | |  |  |
| FOLFOX | 1 (1.0%) | | 3 (2.7%) | |  | |  |  |
| FOLFIRI | 45 (45%) | | 38 (34%) | |  | |  |  |
| FOLFIRINOX | 1 (1.0%) | | 0 (0%) | |  | |  |  |
| Trifluridine/tipiracil | 0 (0%) | | 2 (1.8%) | |  | |  |  |
| Raltitrexed | 1 (1.0%) | | 1 (0.9%) | |  | |  |  |
| **IV-MoAb** | 41 (41%) | | 33 (29%) | | 0.062 | |  |  |
| **IV-MoAb regimen** |  | |  | | **0.007** | |  |  |
| Aflibercept | 0 (0%) | | 1 (3.0%) | |  | |  |  |
| Bevacizumab | 22 (54%) | | 25 (76%) | |  | |  |  |
| Cetuximab | 10 (24%) | | 7 (21%) | |  | |  |  |
| Panitumumab | 9 (22%) | | 0 (0%) | |  | |  |  |
| **HAIC regimen** |  | |  | | >0.9 | |  |  |
| 5FU | 16 (16%) | | 21 (18%) | |  | |  |  |
| Oxaliplatin | 80 (81%) | | 89 (78%) | |  | |  |  |
| Mitomycin | 2 (2%) | | 2 (2%) | |  | |  |  |
| Other | 1 (1%) | | 2 (2%) | |  | |  |  |
| ^1^Median (Q1, Q3); n (%) | | | | | | |  |  |
| ^2^Wilcoxon rank sum test for continuous variables; Fisher's exact test for categorical variables. | | | | | | |  |  |

**Supplementary Table 2: Patient-level description of toxicities leading to HAIC regimen switch.** The main toxicity was defined as the highest-grade adverse event (CTCAE v5.0); when multiple events shared the same grade, all were reported. Concomitant toxicities, treatment setting, and subsequent regimen are shown.

| **Setting** | **Main toxicity leading to switch** | **Other relevant toxicities** | **Subsequent regimen** |
| --- | --- | --- | --- |
| Intensification | asthenia G2, neutropenia G2, abdominal pain G2, neurotoxicity G2, ulcers G2, mucositis G2 | anemia G1, dysgeusia G1, nausea G1, thrombocytopenia G1 | 5-FU |
| Intensification | asthenia G2, abdominal pain G2, diarrhea G2 | alopecia G1, mucositis G1, nausea G1, neurotoxicity G1, skin toxicity G1 | 5-FU |
| Intensification | asthenia G2, thrombocytopenia G2, neurotoxicity G2, allergy G2 | abdominal pain G1, arterial thrombosis G1, nausea G1, portal hypertension | 5-FU |
| Intensification | PAC infection | asthenia G1, anemia G1, thrombocytopenia G1, nausea G1, neurotoxicity G1 | 5-FU |
| Salvage | asthenia G1, abdominal pain G1, allergy G1 |  | Mitomycin |
| Salvage | asthenia G2, neutropenia G2, neurotoxicity G2, allergy G2 | diarrhea G1, nausea G1, thrombocytopenia G1 | 5-FU |
| Salvage | asthenia G2, diarrhea G2, neurotoxicity G2 | allergy G1, anemia G1, nausea G1 | 5-FU |
| Salvage | asthenia G2, anemia G2, thrombocytopenia G2, vomiting G2, diarrhea G2, neurotoxicity G2 | extravasation, nausea G1 | 5-FU |
| Salvage | asthenia G3 | abdominal pain G2, neutropenia G2, anemia G1, neurotoxicity G1 | 5-FU |
| Intensification | abdominal pain G3 | nausea G2, anemia G1, asthenia G1, neurotoxicity G1, skin toxicity G1 | Mitomycin |
| Intensification | abdominal pain G3 | nausea G2, neurotoxicity G2, asthenia G1, thrombocytopenia G1, vomiting G1 | 5-FU |
| Intensification | abdominal pain G3, cholecystitis G3 | allergy G1, asthenia G1, nausea G1, neurotoxicity G1, thrombocytopenia G1, vomiting G1 | 5-FU |
| Intensification | abdominal pain G3 | allergy G1, asthenia G1, nausea G1, vomiting G1 | 5-FU |
| Salvage | abdominal pain G3, neurotoxicity G3 | PAC dysfunction | 5-FU |
| Intensification | neurotoxicity G3 | asthenia G2, dysgeusia G1, nausea G1, skin toxicity G1 | 5-FU |
| Intensification | neurotoxicity G2 |  | 5-FU |
| Intensification | neurotoxicity G2 | asthenia G1, nausea G1, neutropenia G1, thrombocytopenia G1 | 5-FU |
| Salvage | neurotoxicity G2 | allergy G1, anemia G1, diarrhea G1, nausea G1, PAC dysfunction, thrombocytopenia G1 | 5-FU |
| Salvage | neurotoxicity G2 | anemia G1 | 5-FU |
| Intensification | allergy G3, arterial thrombosis | abdominal pain G2, asthenia G1, nausea G1, vomiting G1 | 5-FU |
| Intensification | allergy G2 | abdominal pain G1, anemia G1, asthenia G1 | 5-FU |
| Salvage | allergy G2 | anemia G1, asthenia G1, diarrhea G1, neurotoxicity G1 | 5-FU |
| Salvage | allergy G3 | neurotoxicity G2, skin toxicity G2, asthenia G1, diarrhea G1, nausea G1 | 5-FU |
| Intensification | nausea G2, diarrhea G2, neurotoxicity G2, arterial thrombosis, hemorrhage | asthenia G1 | 5-FU |
| Salvage | nausea G2, vomiting G2, diarrhea G2, allergy G2 | alopecia G1, asthenia G1, neurotoxicity G1 | 5-FU |
| Salvage | neutropenia G3 | asthenia G2, mucositis G2, neurotoxicity G2, anemia G1, nausea G1, thrombocytopenia G1 | 5-FU |
| Salvage | neutropenia G3 | allergy G2, neurotoxicity G2, skin toxicity G2, thrombocytopenia G2, asthenia G1 | 5-FU |
| Salvage | skin toxicity G3 | abdominal pain G2, asthenia G2, neurotoxicity G2, anemia G1, diarrhea G1, nausea G1 | 5-FU |
| Salvage | thrombocytopenia G2, neurotoxicity G2 | abdominal pain G1, asthenia G1, diarrhea G1, dysgeusia G1, nausea G1, neutropenia G1, PAC infection, skin toxicity G1, transaminase increase G1 | 5-FU |

**Supplementary Table *3*. Factors associated with PFS and OS in univariate and multivariable analyses in the HAIC Intensification group.** Significant p-value are highlighted in bold. Acronyms: PFS=progression-free survival; OS=overall survival; ECOG PS= eastern cooperative oncology group performance status; IV-MoAb= intravenous monoclonal antibody.

|  |  | **Univariate analyses** | | | | **Multivariable model for PFS** | | | **Multivariable model for OS** | | |
| --- | --- | --- | --- | --- | --- | --- | --- | --- | --- | --- | --- |
|  |  | **Endpoint** | **HR** | **CI 95%** | **p.value** | **HR** | **CI 95%** | **p.value** | **HR** | **CI 95%** | **p.value** |
| **Age > 65 years** |  | PFS | 0.75 | 0.48 - 1.17 | 0.20 |  |  |  |  |  |  |
|  |  | OS | 0.9 | 0.56 - 1.47 | 0.69 |  |  |  |  |  |  |
| **Female gender** |  | PFS | 0.93 | 0.6 - 1.44 | 0.75 |  |  |  |  |  |  |
|  |  | **OS** | **0.65** | **0.4 - 1.07** | **0.09** |  |  |  | 0.48 | 0.2 – 1.08 | 0.08 |
| **ECOG-PS >1** |  | **PFS** | **3.9** | **1.17 – 13.1** | **0.03** | 0.37 | 0.03 – 4.05 | 0.41 |  |  |  |
|  |  | **OS** | **6.6** | **1.97 – 22.2** | **0.002** |  |  |  | 4.7 | 0.5 – 44.2 | 0.17 |
| **Distal primary tumor** |  | PFS | 1.11 | 0.65 - 1.91 | 0.7 |  |  |  |  |  |  |
|  |  | OS | 0.64 | 0.37 - 1.13 | 0.13 |  |  |  |  |  |  |
| **Primary tumor surgery** |  | PFS | 0.86 | 0.51 - 1.44 | 0.56 |  |  |  |  |  |  |
|  |  | **OS** | **0.48** | **0.28 - 0.83** | **0.008** |  |  |  | 0.7 | 0.2 – 2.3 | 0.57 |
| ***RAS* mutation** |  | **PFS** | **1.72** | **1.08 - 2.73** | **0.02** | 1.49 | 0.75 – 2.95 | 0.26 |  |  |  |
|  |  | **OS** | **1.96** | **1.16 - 3.32** | **0.01** |  |  |  | 1 | 0.4 – 2.5 | 0.99 |
| ***BRAF* mutation** |  | **PFS** | **3.04** | **1.31 - 7.05** | **0.009** | **8.8** | **1.09 – 71.2** | **0.04** |  |  |  |
|  |  | OS | 1.88 | 0.84 - 4.19 | 0.12 |  |  |  |  |  |  |
| **Prior oxaliplatin progression** |  | **PFS** | **3.02** | **1.7 - 5.37** | **<0.001** | 1.71 | 0.66 – 4.4 | 0.26 |  |  |  |
|  |  | **OS** | **4.13** | **2.28 - 7.47** | **<0.001** |  |  |  | **4.9** | **1.6 – 14.67** | **0.004** |
| **Prior line of treatment** |  | **PFS** | **2.22** | **1.24 - 3.97** | **0.007** | 1.47 | 0.67 – 3.24 | 0.34 |  |  |  |
|  |  | OS | 1.5 | 0.79 - 2.88 | 0.21 |  |  |  |  |  |  |
| **Extra-hepatic disease** |  | PFS | 1.37 | 0.83 - 2.28 | 0.22 |  |  |  |  |  |  |
|  |  | OS | 1.51 | 0.86 - 2.66 | 0.15 |  |  |  |  |  |  |
| **Liver burden** | **26-50%** | PFS | 0.63 | 0.35 - 1.15 | 0.13 | 1.3 | 0.52 – 3.25 | 0.57 |  |  |  |
|  | **51-75%** | PFS | 0.74 | 0.37 - 1.48 | 0.4 | 1.32 | 0.5 – 3.49 | 0.58 |  |  |  |
|  | **76-100%** | PFS | 0.94 | 0.48 - 1.81 | 0.84 | 1.88 | 0.73 – 4.8 | 0.19 |  |  |  |
|  | **26-50%** | OS | 0.56 | 0.27 - 1.14 | 0.11 |  |  |  | 0.49 | 0.17 – 1.4 | 0.19 |
|  | **51-75%** | OS | 1.18 | 0.55 - 2.52 | 0.68 |  |  |  | 1.97 | 0.59 – 6.58 | 0.27 |
|  | **76-100%** | **OS** | **1.78** | **0.92 - 3.42** | **0.08** |  |  |  | 1.54 | 0.46 – 5.13 | 0.48 |
| **Oxaliplatin HAIC** |  | **PFS** | **0.4** | **0.23 - 0.7** | **0.001** | **0.23** | **0.08 – 0.69** | **0.008** |  |  |  |
|  |  | **OS** | **0.62** | **0.35 - 1.1** | **0.1** |  |  |  | 0.8 | 0.3 – 2.22 | 0.69 |
| **IV-MoAb** |  | PFS | 0.72 | 0.47 - 1.12 | 0.15 |  |  |  |  |  |  |
|  |  | OS | 0.84 | 0.51 - 1.38 | 0.5 |  |  |  |  |  |  |
| **Baseline CEA > 22ng/mL (median)** |  | PFS | 0.88 | 0.55 - 1.42 | 0.61 |  |  |  |  |  |  |
|  |  | OS | 1.47 | 0.86 - 2.52 | 0.16 |  |  |  |  |  |  |
| **Baseline CA19.9 > 54U/mL (median)** |  | PFS | 1.4 | 0.8 - 2.35 | 0.15 |  |  |  |  |  |  |
|  |  | **OS** | **1.91** | **1.08 - 3.37** | **0.02** |  |  |  | 1.36 | 0.53 – 3.48 | 0.52 |

**Supplementary Table *4*. Factors associated with PFS and OS in univariate and multivariable analyses in the HAIC Salvage group.** Significant p-value are highlighted in bold. Acronyms: PFS=progression-free survival; OS=overall survival; ECOG PS= eastern cooperative oncology group performance status; IV-MoAb= intravenous monoclonal antibody.

|  | **Univariate analysis** | | | | **Multivariable model for PFS** | | | **Multivariable model for OS** | | |
| --- | --- | --- | --- | --- | --- | --- | --- | --- | --- | --- |
|  | **Endpoint** | **HR** | **CI 95%** | **p.value** | **HR** | **CI 95%** | **p.value** | **HR** | **CI 95%** | **p.value** |
| **AGE > 65** | PFS | 0.76 | 0.49 - 1.16 | 0.2 |  |  |  |  |  |  |
| **AGE > 65** | OS | 0.94 | 0.61 – 1.45 | 0.79 |  |  |  |  |  |  |
| **SEX female** | PFS | 0.82 | 0.54 - 1.26 | 0.37 |  |  |  |  |  |  |
| **SEX female** | OS | 0.92 | 0.59 - 1.42 | 0.7 |  |  |  |  |  |  |
| **ECOG PS >1** | **PFS** | **2.25** | **1.27 - 4** | **0.006** | **2.5** | **1.05 – 5.96** | **0.04** |  |  |  |
| **ECOG PS >1** | **OS** | **3.77** | **2.08 - 6.84** | **<0.001** |  |  |  | 2.14 | 0.86 – 5.29 | 0.1 |
| **Distal primary tumor** | PFS | 0.81 | 0.51 - 1.29 | 0.38 |  |  |  |  |  |  |
| **Distal primary tumor** | **OS** | **0.67** | **0.41 - 1.09** | **0.1** |  |  |  |  |  |  |
| **Primary tumor surgery** | PFS | 1.29 | 0.8 – 2.08 | 0.3 |  |  |  |  |  |  |
| **Primary tumor surgery** | OS | 1 | 0.61 - 1.64 | 0.99 |  |  |  |  |  |  |
| ***RAS* mutated** | PFS | 1.2 | 0.79 - 1.85 | 0.39 |  |  |  |  |  |  |
| ***RAS* mutated** | OS | 1.03 | 0.67 - 1.6 | 0.87 |  |  |  |  |  |  |
| ***BRAF* mutated** | PFS | 1.24 | 0.3 - 5.13 | 0.76 |  |  |  |  |  |  |
| ***BRAF* mutated** | OS | 1.53 | 0.21 - 11.22 | 0.68 |  |  |  |  |  |  |
| **Prior oxaliplatin progression** | PFS | 1.34 | 0.88 - 2.03 | 0.17 |  |  |  |  |  |  |
| **Prior oxaliplatin progression** | OS | 1.18 | 0.78 - 1.81 | 0.43 |  |  |  |  |  |  |
| **>2 prior lines of treatments (HAIC >3rd line)** | **PFS** | **1.55** | **1.01 - 2.39** | **0.04** | 1.25 | 0.66 – 2.39 | 0.49 |  |  |  |
| **>2 prior lines of treatments (HAIC >3rd line)** | OS | 1.31 | 0.85 – 2.02 | 0.2 |  |  |  |  |  |  |
| **Extra-hepatic disease** | PFS | 1.2 | 0.81 - 1.81 | 0.36 |  |  |  |  |  |  |
| **Extra-hepatic disease** | **OS** | **1.76** | **1.16 - 2.67** | **0.008** |  |  |  | 1.53 | 0.82 – 2.8 | 0.18 |
| **Liver burden 26-50%** | PFS | 1.72 | 0.86 - 3.45 | 0.13 | 1.9 | 0.66 – 5.48 | 0.24 |  |  |  |
| **Liver burden 51-75%** | PFS | 1.08 | 0.56 – 2.09 | 0.81 | 1.04 | 0.4 – 2.65 | 0.93 |  |  |  |
| **Liver burden 76-100%** | **PFS** | **1.98** | **1.05 - 3.71** | **0.03** | 1.32 | 0.5 – 3.47 | 0.57 |  |  |  |
| **Liver burden 26-50%** | OS | 1.72 | 0.82 - 3.62 | 0.15 |  |  |  | 1.8 | 0.67 – 4.8 | 0.24 |
| **Liver burden 51-75%** | OS | 0.79 | 0.41 - 1.53 | 0.48 |  |  |  | 1.2 | 0.55 – 2.66 | 0.64 |
| **Liver burden 76-100%** | **OS** | **2.34** | **1.23 - 4.45** | **0.009** |  |  |  | **2.6** | **1.1 – 6.05** | **0.03** |
| **Oxaliplatin HAIC** | PFS | 0.94 | 0.57 - 1.57 | 0.82 |  |  |  |  |  |  |
| **Oxaliplatin HAIC** | OS | 0.83 | 0.5 - 1.39 | 0.48 |  |  |  |  |  |  |
| **IV-MoAb** | **PFS** | **0.64** | **0.4 – 1.04** | **0.07** | 0.69 | 0.36 – 1.33 | 0.27 |  |  |  |
| **IV-MoAb** | OS | 1.02 | 0.64 - 1.63 | 0.94 |  |  |  |  |  |  |
| **CEA > 158ng/mL (median salvage CEA)** | **PFS** | **1.58** | **1 - 2.52** | **0.05** | **2.28** | **1.14 – 5.57** | **0.02** |  |  |  |
| **CEA > 158ng/mL (median salvage CEA)** | **OS** | **1.74** | **1.07 - 2.85** | **0.03** |  |  |  | 1.03 | 0.54 – 1.96 | 0.93 |
| **CA19.9 > 167U/mL (median CA19.9)** | PFS | 1.19 | 0.73 - 1.95 | 0.48 |  |  |  |  |  |  |
| **CA19.9 > 167U/mL (median CA19.9)** | **OS** | **1.84** | **1.1 – 3.07** | **0.02** |  |  |  | **2.5** | **1.37 – 4.6** | **0.003** |

**Supplementary Table 5. Multivariable logistic regression analysis of factors associated with PAC-related adverse events (excluding abdominal pain).** The model included concomitant antiangiogenic therapy, prior antiangiogenic exposure, performance status, and HAIC treatment setting. Acronyms: OR=Odds Ratio; CI=95% Confidence intervals

| **Variable** | **OR** | **CI** | **p_value** |
| --- | --- | --- | --- |
| Concomitant antiangiogenic therapy | **2.50** | **(1.24–5.05)** | **0.010** |
| Prior antiangiogenic exposure | 1.45 | (0.68–3.17) | 0.343 |
| Performance status (0-1 vs ≥2) | 0.92 | (0.3–2.52) | 0.874 |
| HAIC setting (intensification vs salvage) | 1.26 | (0.6–2.65) | 0.543 |
